# The effects of shift-work schedules on the sleep, health, safety, and quality of life of police employees during the COVID-19 pandemic

**DOI:** 10.3389/fpsyg.2023.1128629

**Published:** 2023-04-17

**Authors:** Lois James, Stephen James, Loren Atherley

**Affiliations:** ^1^College of Nursing, Health Sciences Campus, Washington State University, Spokane, WA, United States; ^2^Elson S. Floyd College of Medicine, Health Sciences Campus, Washington State University, Spokane, WA, United States; ^3^Performance, Analytics and Research Unit, Seattle Police Department, Seattle, WA, United States

**Keywords:** shift work, police employees, sleep, health, safety, quality of life, COVID-19 pandemic

## Abstract

**Introduction:**

The negative health and safety consequences of police fatigue are increasingly recognized as a critical problem. This study’s objective was to measure the effects of different shift schedules on police employee health, safety, and quality of life.

**Methods:**

A cross sectional research design surveyed employees (*N* = 319) from a large, U.S. west coast municipal police service during the fall of 2020. The survey was made up of a battery of validated instruments designed to assess dimensions of health and wellness (e.g., sleep, health, safety, and quality of life).

**Results:**

We found 77.4% of police employees had poor sleep quality, 25.7% had excessive daytime sleepiness, 50.2% had PTSD symptoms, 51.9% had depressive symptoms, and 40.8% had anxiety symptoms. Working night shifts significantly decreased sleep quality and increased excessive sleepiness. Furthermore, employees working night shifts were significantly more likely to report falling asleep at the wheel while driving home than employees working other shifts.

**Discussion:**

Our findings have implications for interventions designed to promote police employee sleep health, quality of life, and worker safety. We urge researchers and practitioners alike to target night shift workers, to help mitigate these risks.

## 1. Introduction

For several decades, fatigue has been recognized as a critical problem within policing (Vila, 2000). Fatigue resulting from insufficient sleep is associated with a multitude of health, safety, wellness, and performance risks. Research has consistently demonstrated that fatigue resulting from sleep loss, extended wakefulness, shift work, and long work hours impairs decision making (Vila, 2000; Rajaratnam et al., 2011; Waggoner et al., 2012; Satterfield and Van Dongen, 2013; Chen et al., 2016; Andrei et al., 2020; James et al., 2022). Among shift workers, police are some of the worst affected, with the majority suffering from constant fatigue and elevated risk for accidents and errors (Rajaratnam et al., 2011; James et al., 2022). In a seminal study in this area, the AAA Foundation for Traffic Safety (2014) that across a survey of over 2,000 U.S. and Canadian officers, 91% reported routine fatigue, 85% reported driving while drowsy, and an alarming 39% reported falling asleep at the wheel.

Further contributing to fatigue, a large proportion of officers are required to work night shifts, which are counter to their natural circadian rhythms that promote sleep at night (Dement and Vaughan, 1999; James S.M. et al., 2017; Peterson et al., 2019). This “circadian misalignment” results in less overall sleep obtained and greater sleepiness and fatigue during waking hours (James L. et al., 2017). Working night shifts has been found to be especially detrimental to police driving safety. Experimental research from Washington State University (WSU) found that night shift officers were severely impaired and at a significantly higher risk of collision (OR = 2.5) compared to day shift officers when tested in a driving simulator (James and Vila, 2015). To illustrate, research has compared impairments to hand-eye coordination, cognitive performance, task accuracy, and alertness from hours awake to percent blood-alcohol content (BAC) and found that being awake for 17–19 h is the equivalent of having a 0.05 percent BAC and being awake for 24 h is equivalent to a 0.10 percent BAC (Dawson and Reid, 1997; Williamson and Feyer, 2000).

In addition to safety consequences, shift work and resulting sleep restriction and fatigue has critical implications for the long-term health and quality of life of police officers. For example, increased risk for cardio-vascular, gastro-intestinal, and metabolic diseases (James S.M. et al., 2017; Berent et al., 2022), in particular for night or rotating shift workers (Caruso et al., 2006). Police officers are more likely to suffer from these diseases than other workers (Tuttle et al., 2019), in addition to disproportionately suffering from sleep disorders (Vila, 2000; Vila, 2006; Rajaratnam et al., 2011; James et al., 2022), impaired domestic relationships and reduced quality of life (James et al., 2022). Police officers are also at increased risk for developing psychological disorders, such as post-traumatic stress disorder (Kposowa, 1999), substance abuse disorder (Violanti et al., 2013), and increased risk for suicide (Charbonneau, 2000; Tuttle et al., 2019).

Despite the recognition of a fatigue problem within policing many unanswered questions remain. The specific effects of shift schedules on police employee sleep, health, safety, and quality of life have yet to be comprehensively examined across a large police service in the COVID-19 pandemic era. The current study’s objective was to measure these effects in a sample of officers approximately six months into the COVID-19 pandemic. In doing so, we investigated the following research hypotheses related to sleep (H1-2), safety (H3), health (H4-6), and quality of life (H7):

*H1*: Police employees working night shifts will have significantly worse sleep quality than those working day or evening shifts.

*H2*: Police employees working night shifts will be significantly sleepier than those working day or evening shifts.

*H3*: Police employees working night shifts will be at increased safety risk of falling asleep at the wheel driving home than those working day or evening shifts.

*H4*: Police employees working night shifts will have significantly higher risk of PTSD than those working day or evening shifts.

*H5*: Police employees working night shifts will have significantly higher rates of depression than those working day or evening shifts.

*H6*: Police employees working night shifts will have significantly higher rates of anxiety than those working day or evening shifts.

*H7*: Police employees working night shifts will have significantly lower quality of life than those working day or evening shifts.

We believe this work fills a critical gap in the research literature and could provide timely and nationally relevant information on a leading problem within policing.

## 2. Materials and methods

### 2.1. Research design, sample, and procedures

We used a cross sectional research design to survey employees (*n* = 419) from a large municipal police service, on the west coast of the U.S. during the fall of 2020. At the time of testing, the service had approximately 1,150 deployable^1^ sworn employees, all of whom were surveyed. No exclusion criteria were specified. 419 responded, with 319 filling in survey questions completely (allowing for generation of global/total scores for survey instruments), resulting in a total response rate of 36% with 27% providing complete responses. This project was approved by the (blinded for anonymity) Institutional Review Board prior to participant recruitment. The observations reported here were generated as part of a larger ongoing National Institute of Justice (NIJ) funded, longitudinal mixed-methods study exploring the effects of fatigue on health, safety, wellness, and performance within the police service.

A link to the secure online survey was emailed to all police employees *via* service email, with a request to participate. The survey remained open for a month-long period (September 1st to October 1st) during which time weekly reminders were emailed to employees. When employees clicked on the link to the survey, they were directed to a consent form that explained the purpose of the study, participant expectations and burdens, and any potential risks or benefits. The survey could not be accessed without signing the consent form electronically.

Characteristics of our sample are depicted in Table 1. During the month of October 2020, the agency employed approximately 1,950 employees, of which 67% were sworn police officers and the remainder (33%) a mix of civilian FTE and temporary civilian (not sworn). Just over 75% of our respondents were sworn officers, slightly higher than the service makeup (the remainder were civilian staff). A majority of the sample was white (72%) and male (67%). The modal age range was, 35–44,^2^ and 83% of the sample were between 25–54. The average years of experience within the police service was 12 (SD = 5). 29% of the sample had prior military experience.

**Table 1:** Characteristics of the sample (*N* = 319).

		Frequency	Percent
Race	American Indian	1	0.3
	Asian	24	7.6
	Black	17	5.4
	Hispanic	20	6.4
	White	226	72
	Other	26	8.3
Gender	Female	102	32.3
	Male	210	66.5
	Other	4	1.3
Age	Under 25	3	1
	25–34	60	19.1
	35–44	115	36.6
	45–54	87	27.7
	55–64	42	13.4
	Over 65	7	2.2
Job Type	Civilian	78	24.5
	Sworn	241	75.5
Prior Military	Yes	69	29
	No	169	71
Shift	3 am–11 am	33	13.8
	11 am–8 pm	39	16.3
	8 pm–3 am	39	16.3
	9 am–5 pm	87	36.4
	Other	30	17.2
		Mean	Std. Deviation
Years of Experience		11.56	5.478

About 36% of the sample worked a standard 8-h day shift (9 am–5 pm). Civilian staff largely fell into this category, as well as executive level employees. Sworn personnel predominantly worked one of the following: 14% worked night shift (3 am–11 am), 16% worked day shift (11 am–8 pm), and 16% worked evening shift (8 pm–3 am). The remainder of the sample worked some other shift schedule (for example one division in the service worked rotating shifts). Shift length typically ranged from 7–10 h.

### 2.2. Materials

A battery of validated, commonly accepted instruments designed to assess multiple dimensions of sleep, safety, health, and quality of life. Instruments were the Pittsburg Sleep Quality Index – PSQI (Buysse et al., 1989), the Epworth Sleepiness Scale – ESS (Johns, 1994), the PTSD Checklist for DSM-5 – PCL-5 (Weathers et al., 2013), the Patient Health Questionnaire-9 – PHQ-9 (Kroenke and Spitzer, 2002), the General Anxiety Disorder-7 – GAD-7 (Spitzer et al., 2006), and the World Health Organization Quality of Life – WHOQOL (Bonomi et al., 2000).

In brief, the PSQI is a 19-item survey with seven subcategories: subjective sleep quality, sleep latency, sleep duration, habitual sleep efficiency, sleep disturbances, use of sleeping medication, and daytime dysfunction. Global scores (summed across categories) range from 0–21 and a clinical cutoff of 5 is recommended for identifying poor sleep quality. Cronbach’s alpha for the PSQI is 0.83.

The ESS is an 8-item scale in which respondents’ rate on a 4-point scale (0–3) their likelihood of falling asleep while engaged in eight different activities (including falling asleep while driving). The total ESS score can range from 0 to 24. The higher the score, the higher that their propensity for falling asleep during waking hours, or “daytime sleepiness,” with a score of 11 or over being used to identify “excessive daytime sleepiness.” Cronbach’s alpha for the ESS is 0.82.

The PCL-5 is a 20-item survey measuring symptoms of PTSD. Scores range from 0–80, with a cutoff range of 31–33 being indicative of PTSD. The PCL-5, although frequently used as part of PTSD diagnosis, is not a diagnostic tool in and of itself. Cronbach’s alpha for the PCL-5 is 0.84.

The PHQ-9 is a nine-item scale measuring depression. Scores range from 0–27, where 0–4 points equals minimal depression, 5–9 points indicates mild depression, 10–14 points indicates moderate depression, 15–19 points indicates moderately severe depression, and 20 or more points indicates severe depression. Cronbach’s alpha for the PHQ-9 is 0.89.

The GAD-7 is a seven-item scale designed to assess anxiety. Scores range from 0–21, with 0–4 indicating minimal anxiety, 5–9 mild anxiety, 10–14 moderate anxiety, and greater than 15 indicating severe anxiety. Cronbach’s alpha for the GAD-7 is 0.83.

Finally, the WHOQOL is a quality-of-life assessment developed by the World Health Organization. In this study we used the brief version, which is a 26-item instrument consisting of four domains: physical health (7 items), psychological health (6 items), social relationships (3 items), and environmental health (8 items); and two items rating satisfaction with general health and quality of life. Cronbach’s alpha for the WHOQOL brief version is 0.89.

### 2.3. Analyses

First, descriptive statistics were used to provide means, standard deviations, and frequencies for global survey scores, and investigate trends within our data.

Second, linear and ordinal regressions models were used to test our research hypotheses. Regression is an appropriate technique for analyzing the ability of one or more independent variables to predict a dependent variable. Separate linear models were run for sleep quality, sleepiness, post-traumatic stress, depression, and anxiety. The “falling asleep at the wheel” variable and the “quality-of-life” variable were both rated on an ordinal scale, requiring ordinal regression models. Dummy variables were created for each category within the independent variable: day shift (1 = yes, 0 = no), evening shift (1 = yes, 0 = no), night shift (1 = yes, 0 = no), with day shift acting as reference (thus not entered into models).

## 3. Results

Overall, the sample had substantial sleep, sleepiness, PTSD, depression, and anxiety problems. Table 2 depicts average scores for each instrument. When considering cutoffs for each scale, 77.4% had poor sleep quality, 25.7% had excessive daytime sleepiness, 50.2% had PTSD symptoms (with 42.9% having moderate to severe symptoms). 51.9% of the sample showed symptoms of depression (35.3% had mild depressive symptoms, 9.7% had moderate depressive symptoms, and 6.9% had severe depressive symptoms). 40.8% of the sample reported symptoms of anxiety (22.6% had mild anxiety symptoms, 10.9% had moderate anxiety symptoms, and 7.3% had severe anxiety symptoms).

**Table 2:** PSQI (sleep quality), ESS (daytime sleepiness), PCL-C (PTSD symptoms), PHQ-9 (depression), GAD-7 (anxiety) scores within the sample (*N* = 319).

	Min	Max	Mean	SD
PSQI Score	1	20	8.59	3.78
ESS Score	0	24	8.05	4.62
PCL-C Score	17	85	33.56	14.25
PHQ-9 Score	0	27	7.38	5.66
GAD-7 Score	0	21	6.7	5.51

When examining health and quality of life ratings within the sample, we found that approximately 30% of the sample rating their health as either poor or very poor (see Table 3 for details). However, only about 10% rated their quality of life as either poor or very poor.

**Table 3:** Health and quality of life ratings with the sample (*N* = 319).

		Frequency	Percent
QOL rating	Very poor	6	1.9
	Poor	26	8.2
	Neither poor nor good	46	14.4
	Good	189	59.2
	Very good	52	16.3
Health satisfaction	Very dissatisfied	5	1.6
	Dissatisfied	87	27.3
	Neither satisfied nor dissatisfied	64	20.1
	Satisfied	144	45.1
	Very satisfied	19	6

For ease of interpretation linear regression models are presented below by hypothesis. Details of models are depicted in Tables 4, 5.

**Table 4:** Results from linear regression models testing the impact of shift type on sleep quality, sleepiness, PTSD, depression, and anxiety.

		*B*	Std. error	Beta	*t*	Sig.
Sleep Quality	Night Shift	2.93	0.77	0.22	3.83	0.001
(PSQI)	Evening Shift	−0.18	0.69	−0.01	0.19	0.85
Sleepiness	Night Shift	3.7	0.83	0.24	4.43	0.001
(ESS)	Evening Shift	0.26	0.69	0.02	0.34	0.74
PTSD	Night Shift	3.4	2.91	0.07	1.17	0.24
(PCL-5)	Evening Shift	0.61	2.78	0.1	0.22	0.83
Depression	Night Shift	1.3	1.12	0.07	1.17	0.24
(PHQ-9)	Evening Shift	−0.31	1.07	−0.02	0.19	0.83
Anxiety	Night Shift	0.61	1.15	0.03	0.53	0.6
(GAD-7)	Evening Shift	−0.53	1.09	−0.03	0.48	0.63

**Table 5:** Results from ordinal regression models testing the impact of shift type on likelihood of falling asleep at the wheel and quality of life rating.

		Estimate	Std. Error	Wald	*df*	Sig.
Falling Asleep at the Wheel	Night Shift	1.52	0.39	14.3	1	0.001
(ESS question)	Evening Shift	0.44	0.44	1.02	1	0.31
Quality of Life	Night Shift	0.36	0.36	0.99	1	0.32
(WHOQOL Question)	Evening Shift	0.7	0.33	4.47	1	0.08

*H1*: Police employees working night shifts will have significantly worse sleep quality than those working day or evening shifts.

The model testing the impact of shift schedule on sleep quality (PSQI scores) was significant (*f* = 7.52; *df* = 2,280; *p* < 0.05), with shift explaining 5% of the variance in sleep quality. Within the model, working night shifts was significant (*B* = 2.93, *p* < 0.001), with employees working nights having an average PSQI score of 11.27 (SD = 4.13) compared to employees not working nights having an average PSQI score of 8.17 (SD = 3.66). In light of these results, our first research hypothesis is supported.

*H2*: Police employees working night shifts will be significantly sleepier than those working day or evening shifts.

The model testing the impact of shift schedule on sleepiness (ESS scores) was also significant (*f* = 9.86; *df* = 2,318; *p* < 0.01), with shift explaining 6% of the variance in sleepiness. Working night shifts was significant (*B* = 3.70, *p* < 0.001), with employees working nights having an average ESS score of 11.33 (SD = 4.82) compared to employees not working nights having an average ESS score of 7.67 (SD = 4.46). Our second research hypothesis is also supported.

*H3*: Police employees working night shifts will be at increased safety risk of falling asleep at the wheel driving home than those working day or evening shifts.

The model testing the impact of shift on the specific question of the ESS asking about likelihood of falling asleep at the wheel found that night shift employees were significantly more likely to report falling asleep at the wheel driving home than employees working other shifts (*Chi^2^* = 15.30.93; *df* = 2; *p* < 0.001), with shift explaining 5% of the variance in likelihood of falling asleep at the wheel. Our third research hypothesis is supported.

*H4*: Police employees working night shifts will have significantly higher risk of PTSD than those working day or evening shifts.

Overall, the model testing the impact of shift schedule on PTSD risk (PCL-5 scores) was not significant at the *p* < 0.05 level, with shift explaining less than 2% of the variance in PTSD risk. Although employees working nights had higher average PCL-5 scores (37 compared to 33 – notably putting them on average over the cutoff for PTSD) the difference was not significant, thus our fourth research hypothesis is not supported.

*H5*: Police employees working night shifts will have significantly higher rates of depression than those working day or evening shifts.

The model testing the impact of shift schedule on depression (PHQ-9 scores) was not significant at the *p* < 0.05 level, with shift explaining less than 1% of the variance in depression. Employees working nights had slightly higher average PHQ-9 scores (8.6 compared to 7.2), however this difference was not significant, thus our fifth research hypothesis is not supported.

*H6*: Police employees working night shifts will have significantly higher rates of anxiety than those working day or evening shifts.

The model testing the impact of shift schedule on anxiety (GAD-7 scores) was not significant at the *p* < 0.05 level, with shift explaining less than 1% of the variance in anxiety. Although employees working nights had slightly higher average GAD-7 scores (7.3 compared to 6.6) the difference was not significant, thus our sixth research hypothesis is not supported.

*H7*: Police employees working night shifts will have significantly lower quality of life than those working day or evening shifts.

The impact of shift schedule on WHOQOL ratings of satisfaction was not significant at the *p* < 0.05 level, with shift explaining less than 1% of the variance in ratings of quality of life. Our final hypothesis is not supported.

## 4. Discussion

Our study goal was to determine the impact of different shift types on police employee sleep, health, safety, and wellness. We found that employees working the night shift were significantly more likely than employees working other shifts to suffer from poor sleep quality and excessive daytime sleepiness. They were also significantly more likely to report falling asleep at the wheel than employees working other shifts—putting them at an increased safety risk. Symptoms of PTSD, depression, anxiety and quality of life were not significantly different based on officer shift. Our results indicate that working night shifts was predominantly detrimental to sleep and driving safety and not to mental health or quality of life.

Our findings support what much of the previous literature attests—that police suffer from considerable health, wellness, and safety risks (Tuttle et al., 2019; James et al., 2022). Generally speaking, the rates of poor sleep, excessive sleepiness, PTSD, depression, and anxiety that we found within the sample are higher than previously estimated (Charbonneau, 2000; Violanti et al., 2013). This suggests that policing during the COVID-19 pandemic is particularly fatiguing and stressful.

The specific risks associated with working night shifts to sleep quality and excessive sleepiness are likely due to a combination of sleep restriction and circadian misalignment associated with sleep and wake schedules that are not aligned with humans’ natural circadian rhythms. Our sleep quality and sleepiness results echo previous research that has found night shift work to be the most challenging from a sleep health perspective (James et al., 2020). Our finding that night shift employees were at greater risk of falling asleep while driving is also consistent with the previous literature (James and Vila, 2015). Although within our sample different shifts did not significantly predict health and wellness factors such as PTSD, depression, anxiety, or quality of life it is notable that average PTSD scores within the night shift employee group were above the clinical cutoff, whereas PTSD scores for employees working other shifts were not.

Our findings have several implications for policy and intervention. Programs designed to mitigate fatigue, such as sleep hygiene education, on duty napping interventions, or fatigue countermeasures training would be best targeted to night shift officers who experience the worst sleep and sleepiness outcomes. Several agencies have implemented interventions such as these, with consistently positive results (James et al., 2022). Services should also consider offering ride share options for night shift employees to prevent risks associated with falling asleep at the wheel. Research is needed to determine whether an intervention such as this could reduce the safety risks of drowsy driving. The specific mechanisms by which the COVID-19 pandemic impacts police sleep, health, safety and wellness are not yet understood. It is possible that understaffing plays a role, or that fear of contamination adds to stress, which consequently impacts sleep, health, and quality of life. Some qualitative based research has explored these specific mechanisms. For example, Duran and Woodhams (2022) found that police analysts working from home struggled with limited human interaction and lack of agency support. Additional research is needed to understand specific effects on front line or patrol officers. Finally, given the very high rates of mental health concerns uncovered in our study, interventions on mindfulness (for example Yoga), mental illness stigma reduction (for example providing officers with access to “mental health” days as part of sick leave), and peer support (for example peer support systems to discuss mental health concerns), as well as others are clearly warranted.

The results of the current study need to be considered in light of several limitations. First, the effects of overtime or secondary employment were not measured, both of which could impact police employee sleep, health, safety, and quality of life. We urge researchers to collect data on overtime hours in future studies, especially given the rising rates of understaffing in police services nationwide. Second, as is the case with most police research, our sample has limited racial and ethnic diversity (although reflective of the partner service). Third, cross sectional research designs (opposed to longitudinal designs) make specific predictions from regression models challenging due to lack of temporal order. That said, officers within the test service tend to work their assigned shift for a period of 12 months, so we believe using shift schedules to predict sleep, health, safety, and wellness information collected at a single time point is appropriate. Finally, selection bias needs to be acknowledged, with employees who were concerned about fatigue, health, or wellbeing likely being oversampled.

In conclusion, we found that our test service’s employees had startlingly high rates of poor sleep, excessive sleepiness, PTSD, depression, and anxiety. Working the night shift significantly predicted worse sleep quality, more excessive sleepiness, and higher likelihood of falling asleep while driving compared to working day or evening shifts. Our findings have implications for interventions designed to promote police employee sleep, health, quality of life, and worker safety. We urge researchers and practitioners alike to target night shift officers with interventions designed to help mitigate these effects.

## Data availability statement

The raw data supporting the conclusions of this article will be made available by the authors, without undue reservation.

## Ethics statement

The studies involving human participants were reviewed and approved by Washington State University. The patients/participants provided their written informed consent to participate in this study.

## Author contributions

LJ was the principal investigator on the study and was responsible for study design, data analysis, and manuscript drafting. SJ was the co-investigator on the study and was responsible for data collection and manuscript review. LA was the site coordinator on the study and was responsible for participant recruitment and manuscript review. All authors contributed to the article and approved the submitted version.

## Funding

Funded by National Institute of Justice (NIJ) grant #2019-R2-CX-0022.

## Conflict of interest

The authors declare that the research was conducted in the absence of any commercial or financial relationships that could be construed as a potential conflict of interest.

## Publisher’s note

All claims expressed in this article are solely those of the authors and do not necessarily represent those of their affiliated organizations, or those of the publisher, the editors and the reviewers. Any product that may be evaluated in this article, or claim that may be made by its manufacturer, is not guaranteed or endorsed by the publisher.

## Footnotes

^1^The count of deployable officers is calculated by the agency as those sworn employees, of all rank, fully qualified (no student officers) and on active full duty (excluding light duty employees not cleared for field duty due to health or administrative reasons).

^2^The survey asked age category not exact age, so means and standard deviations are not available.
